# MicroRNA-345-3p expression inhibits colorectal cancer liver metastasis by targeting KHDRBS1 and TGF-β signaling pathway

**DOI:** 10.1186/s41065-026-00715-5

**Published:** 2026-07-30

**Authors:** Song Fan, Jian Zhao, Songjie Liu, Bing Xu, Xiangting Cheng

**Affiliations:** 1 Department of Colorectal Hernia Surgery, Binzhou Medical University Hospital, Binzhou, 256600 China; 2 Department of General Surgery, Xinxiang Central Hospital, Xinxiang, 453001 China; 3 Department of Gastrointestinal Surgery, Zibo Central Hospital, No.54 Gongqingtuan West Road, Zhangdian District, Zibo, 250036 China

**Keywords:** MiR-345-3p, Liver metastasis, Colorectal cancer, KHDRBS1, TGF-β signaling pathway

## Abstract

**Objective:**

The aim of this study was to investigate the association of microRNA-345-3p (miR-345-3p) with liver metastasis in colorectal cancer (CRC) and to explore its underlying molecular mechanisms using in vitro approaches.

**Methods:**

Serum specimens were collected from 110 CRC patients and 90 healthy individuals. Quantitative real-time PCR (qRT-PCR) was employed to assess the expression levels of miR-345-3p and KH RNA binding domain containing, signal transduction associated 1 (KHDRBS1) expression. The associations of miR-345-3p with clinical pathological characteristics, survival, and liver metastasis were analyzed. In vitro cell experiments were conducted to validate the effects of miR-345-3p on CRC cell growth and epithelial-mesenchymal transition (EMT). Target genes and signaling pathways were verified using luciferase reporter assays, rescue experiments, and Western blot analysis.

**Results:**

A significant decrease in miR-345-3p expression was observed in CRC patients with liver metastasis. Low levels of miR-345-3p were correlated with advanced tumor node metastasis (TNM) stages, deeper invasion, lymph node metastasis, and poorer prognosis. Overexpression of miR-345-3p inhibited CRC cell growth and suppressed EMT-associated molecular changes. Bioinformatics analyses and experimental results confirmed KHDRBS1 as a direct target gene of miR-345-3p. Furthermore, rescue experiments demonstrated that overexpression of KHDRBS1 reverses the tumor-suppressive effects of miR-345-3p, indicating that miR-345-3p functions by regulating the transforming growth factor-β (TGF-β) signaling pathway.

**Conclusions:**

Decreased circulating levels of miR-345-3p are associated with liver metastasis in CRC. In vitro studies suggest that miR-345-3p may exert its tumor-suppressive effects by targeting KHDRBS1 and suppressing the TGF-β signaling pathway, thereby inhibiting CRC cell migration and invasive capacity.

## Background

As a leading malignant tumor of the gastrointestinal tract, colorectal cancer (CRC) has exhibited an upward trend in incidence over recent years, imposing a significant burden on patient health and life outcomes [1, 2]. The pathogenesis of CRC involves a complex, multi-step progression driven by the interplay of various genetic alterations [3]. Abnormalities cell proliferation, differentiation, apoptosis, and interactions among different genes and signaling pathways are associated with its development [4, 5]. Moreover, an increased risk of CRC has been linked to well-documented risk factors, including dietary and lifestyle habits, genetic predisposition, the presence of polyps, and gene mutations [6, 7].

Epithelial-mesenchymal transition (EMT) refers to the biological process in which epithelial cells transform from an epithelial state to a mesenchymal phenotype [8]. During EMT, a decrease in cell-cell adhesion is observed, accompanied by enhanced motility. Furthermore, these cells acquire the ability to infiltrate adjacent tissues of the primary tumor, enter lymphatic or blood vessels, traverse the circulatory and lymphatic systems, and ultimately establish metastatic sites [9]. Consequently, EMT is implicated in the initiation, invasion, metastasis, and treatment resistance of cancer [10]. Approximately 50% of CRC patients progress to distant metastases, with liver metastasis being the most common site, significantly contributing to patient mortality rates [11, 12]. Recent studies have further elucidated the molecular mechanisms underlying CRC progression and metastasis, involving various signaling pathways and non-coding RNAs, including the ERK/p65/MMP9 pathway [13], the circRNA-miRNA axis [14], the c-Myc/HIF-1α-mediated Warburg effect [15], and the miR-1204/MASPIN network [16]. Currently, the primary treatment for CRC liver metastasis involves either synchronous or staged surgical resection of both the primary intestinal tumor and the liver metastases [17]. At present, there is a lack of highly effective clinical indices for the early prediction of liver metastasis in CRC, nor are there effective therapeutic targets for treating advanced CRC with concurrent metastases.

MicroRNAs (miRNAs) are a class of non-coding RNAs consisting of 22–24 nucleotides. By influencing physiological and pathological activities in tumor cells, miRNAs can function as either oncogenic or tumor-suppressing agents across various cancer types [18, 19]. MicroRNA-345-3p (miR-345-3p) has been shown to be involved in the initiation and development of human tumors, such as hepatocellular carcinoma (HCC) and breast cancer [20, 21]. However, the role of miR-345-3p in CRC remains unexplored. Concurrently, KH RNA binding domain containing, signal transduction associated 1 (KHDRBS1), also known as Sam68, has been reported to contribute to tumor metastasis and progression in several cancers, including CRC [22]. Nonetheless, the precise role of this protein in CRC metastasis and progression requires further investigation.

This present study integrated datasets from public datasets to identify the miR-345-3p/KHDRBS1 axis as a promising regulator of CRC liver metastasis. Furthermore, the expression levels of miR-345-3p and KHDRBS1 were analyzed in serum samples, along with their correlation with patient prognosis. In vitro cellular experiments were conducted to explore the potential mechanisms by which miR-345-3p promotes CRC cell metastasis.

## Methods

### Study subjects

The present study enrolled 110 patients diagnosed with CRC through histopathological examination at Xinxiang Central Hospital. The inclusion criteria were as follows: (1) Patients met the CRC diagnostic criteria, confirmed by surgical pathology; (2) The diagnosis was the first occurrence of primary CRC; (3) Clinical records were complete. The exclusion criteria included: (1) Concurrent malignant tumors; (2) Abnormal liver or kidney function; (3) Autoimmune diseases or active infections. Additionally, none of the CRC patients had received radiotherapy, chemotherapy, or targeted therapy prior to enrolment. A control group of 90 healthy individuals, matched for age and gender and undergoing routine physical examinations, was also selected (Table 1).

**Table 1:** Comparisons of the general information in CRC patients and healthy controls

Factors	Controls (*n* = 90)	CRC (*n* = 110)	*P* values
Age (years)	59.36±10.26	59.16±11.17	0.900
Gender			0.764
Male	48	61	
Female	42	49	
BMI (kg/m^2^)	22.12±1.79	21.93±2.17	0.503

*CRC* colorectal cancer, *BMI* body mass index

*P* < 0.05 indicates a significant difference

Prior to participation, all subjects received a comprehensive explanation of the study protocol and voluntarily signed written informed consent forms. The Ethics Committee of Xinxiang Central Hospital approved this study. All methods were performed in accordance with relevant guidelines, regulations and the Declaration of Helsinki.

Patients with CRC were categorized into two groups based on the presence of liver metastasis (LM) at diagnosis: the non-metastatic group (non-CRLM, *n* = 89) and the metastatic group (CRLM, *n* = 21). General clinical data were collected and organized, including age, gender, tumor location, tumor size, histological differentiation grade, depth of invasion, tumor node metastasis (TNM) stage, and lymph node metastasis (LNM) status. All patients underwent a five-year follow-up to record progression-free survival (PFS). For laboratory sample processing, 5 mL of peripheral venous blood was collected preoperatively. Serum samples were collected and processed according to standardized operating procedures. Following blood collection, samples were allowed to clot at room temperature for 30 min. Serum was then separated by centrifugation at 3000 rpm for 10 min at 4℃. After aspirating the supernatant serum, a second centrifugation was performed to thoroughly remove residual blood cells. The separated serum was immediately aliquoted and maintained at -80℃ for later experimental use.

### Hemolysis screening of serum samples

To ensure the accuracy of miRNA detection, all serum samples undergo rigorous hemolysis assessment prior to RNA extraction. Samples exhibiting obvious hemolysis (deep red) are excluded through visual inspection. Further screening employs spectrophotometric measurement of serum absorbance at 414 nm (A414). Samples meeting the criterion A414 ≤ 0.2 are accepted, while those with A414 > 0.2 are excluded. This threshold was established based on previous studies demonstrating that hemoglobin absorption at 414 nm exceeding 0.2 significantly interferes with serum miRNA quantification [23, 24].

### Quantitative real-time polymerase chain reaction (qRT-PCR) detection

Total RNA was isolated from frozen serum specimens using TRIzol^®^ reagent (Invitrogen, #15596026). RNA concentration and purity were assessed using a NanoDrop 2000 spectrophotometer, ensuring an A260/A280 ratio within the range of 1.8 to 2.0. For miR-345-3p detection, reverse transcription was performed using miRNA-specific stem-loop primers and the TaqMan miRNA Reverse Transcription Kit. For KHDRBS1 mRNA detection, reverse transcription was conducted with the PrimeScript™ RT Kit. qRT-PCR reactions were performed on a QuantStudio 5 Real-Time Fluorescent Quantitative PCR System (Applied Biosystems) using SYBR Green Pro Taq HS Premix Reagent (Accurate Biology, CAT#AG11701, Human, China). U6 snRNA was selected as the endogenous reference for miR-345-3p due to its stable expression in pilot experiments and previous literature support. Glyceraldehyde-3-phosphate dehydrogenase (GAPDH) served as the endogenous gene for KHDRBS1 mRNA. The 2^−ΔΔCt^ equation was utilized for the calculation of gene expression amounts.

### Cell culture and transfection

Human CRC cell lines SW480 (ATCC, CCL-228), HT-29 (ATCC, HTB-38), Caco-2 (ATCC, HTB-37), LoVo (ATCC, CCL-229) were obtained from the American Tissue Culture Collection (ATCC, USA), and normal colonic epithelial cells NCM460 (INCELL, CVCL 0460) was acquired from INCELL corporation (San Antonio, TX, USA). The cells were propagated in high‑glucose DMEM medium (Gibco, Grand Island, NY, USA) enriched with 10% fetal bovine serum (Gibco, Grand Island, NY, USA), and routinely split under a 37 °C, 5% CO₂ atmosphere.

To explore the biological function of miR-345-3p, we designed the following transfection groups: a negative control group (mimic-NC) and a miR-345-3p overexpression group (miR-345-3p mimic). Subsequently, rescue experiments were conducted, which included the miR-345-3p mimic combined with an empty control plasmid group (miR-345-3p mimic + oe-NC) and the miR-345-3p mimic combined with a KHDRBS1 overexpression plasmid group (miR-345-3p mimic + oe-KHDRBS1). Lipofectamine 3000 reagent (Thermo Fisher Scientific, Invitrogen, #L3000015) was utilized in accordance with the manufacturer’s guidelines.

### CCK-8 detection

Logarithmic phase cells were seeded into a 96-well plate and cultured for various time points (0, 24, 48, and 72 h) for detection. After adding 10 µL CCK‑8 (Dojindo, #CK04, Kumamoto, Japan) to each well, the cells were incubated at 37 °C in the dark for 2 h. The absorbance at 450 nm was subsequently measured using a microplate reader.

### Transwell assay

Cell migration and invasion capabilities were assessed using Transwell chambers (Corning company, NY, USA, #3422; 8-µm pore size). For migration assays (without Matrigel coating), transfected cells were resuspended in serum-free medium and 5 × 10^4^ cells in 200 µL were seeded into the upper chamber. For invasion assays, the upper chamber was pre-coated with 50 µL Matrigel (BD Biosciences, CAT#356234, USA) and incubated at 37℃ for 1 h to solidify. Then, 1 × 10^5^ cells in 200 µL serum-free medium were seeded into the Matrigel-coated upper chamber. For both assays, the lower chamber was filled with 600 µl complete medium with 10% FBS as a chemoattractant. After 24 h of incubation at 37℃, cells remaining on the upper surface of the membrane were gently removed with a cotton swab. Cells that had migrated or invaded through the membrane to the lower surface were fixed with 4% paraformaldehyde, stained with 0.1% crystal violet, and quantified by counting five randomly selected fields under a light microscope at 200× magnification.

### Bioinformatics analysis

To explore the potential mechanism of miR-345-3p, bioinformatics methods were employed to predict its downstream target genes. High-confidence candidate target genes were identified by cross-referencing the TargetScan, miRDB, and ENCORI databases. Further functional annotation of the target genes was conducted using an online tool (https://www.bioinformatics.com.cn/) for Gene Ontology (GO) annotation and Kyoto Encyclopedia of Genes and Genomes (KEGG) pathway analysis.

### Luciferase reporter assay

To validate the binding relationship between miR-345-3p and KHDRBS1, a dual luciferase reporter assay was performed. Initially, a wild-type sequence (KHDRBS1-WT) containing the predicted binding site in the KHDRBS1 3’ UTR and constructed a control plasmid (KHDRBS1-MUT) with a mutated binding site was constructed; both were inserted into the pGL3-Basic reporter vector. The reporter plasmids were co-transfected with either a miR-345-3p mimic, a miR-345-3p inhibitor, or their corresponding NCs into CRC cells. Following 48 h of transfection, luciferase activity was measured using the Dual-Luciferase^®^ Reporter Gene Detection System (Promega, Madison, WI, USA, CAT#E1910). Relative luciferase activity was calculated using the Renilla luciferase plasmid as an internal control.

### Western blot

Proteins were extracted from collected cells of each group using RIPA lysis buffer (Beyotime, Shanghai, China, CAT#P0013B). After quantification, equal protein loads were resolved by SDS-PAGE and electroblotted onto polyvinylidene difluoride (PVDF) membranes (Millipore, Billerica, MA, USA, CAT#IPVH00010). Primary antibodies against KHDRBS1 (Proteintech, #10222-1-AP; 1:1000), transforming growth factor-β1 (TGF-β1) (Abcam, #ab215715; 1:1000), E-cadherin (Cell Signaling Technology, #3195; 1:1000), N-cadherin (Cell Signaling Technology, #13116; 1:1000) and GAPDH (Cell Signaling Technology, #5174; 1:5000, internal control) were incubated overnight at 4 °C. Following overnight incubation, membranes were incubated with HRP-conjugated secondary antibodies (Cell Signaling Technology, #7074; 1:2000) at room temperature. Signal detection was performed using enhanced chemiluminescence (ECL) chemiluminescent substrate (Thermo Scientific, #34580, Waltham, MA, USA), and band intensities were quantified with ImageJ software to determine the relative expression of target proteins.

### Statistics

Statistical analysis was carried out with SPSS 26.0, and figures were generated using GraphPad Prism 9.0. Continuous variables are presented as Mean ± standard deviation (SD), and comparisons between groups were made with Student’s t-test or analysis of variance (ANOVA). Categorical variables were evaluated by χ² test. The diagnostic performance of miR-345-5p was conducted using plotting (ROC) curve. Survival outcomes were analyzed with the Kaplan–Meier method and Cox proportional hazards regression. Risk factors for liver metastasis were identified by logistic regression analysis. Using the mean value of relative expression of miR-345-3p as the cutoff, the CRC cohort were stratified into two groups: low miR-345-3p (*n* = 56) and high miR-345-3p (*n* = 54). The mean was selected as the cutoff because it reflects the central tendency of expression levels in the cohort and is a commonly used threshold in exploratory miRNA studies when no clinically predefined cutoff exists. A *P*-value of less than 0.05 was regarded as statistically significant.

## Results

### MiR-345-3p was downregulated in patients with CRLM

**Fig. 1:**
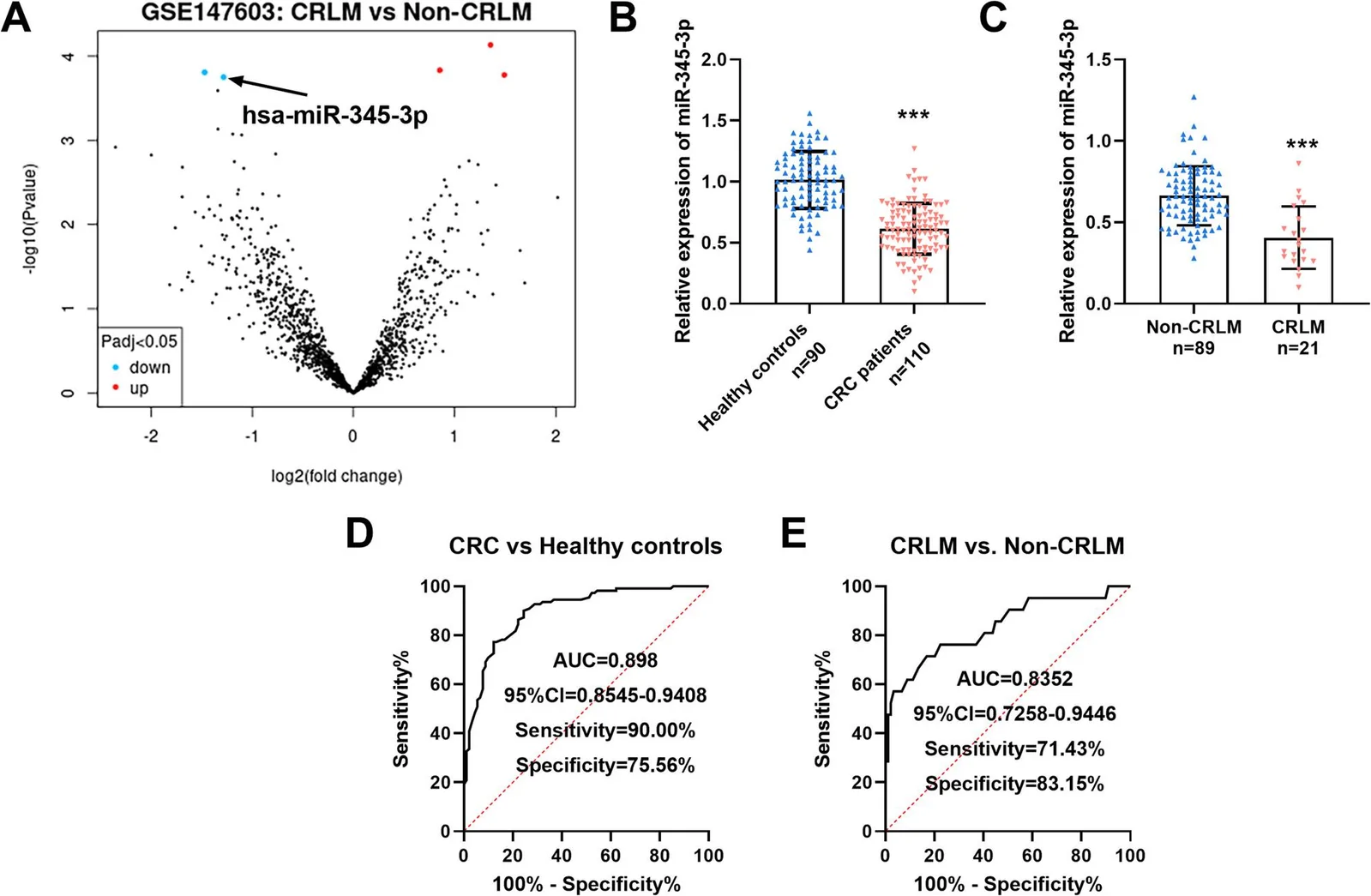
The relative abundances of miR-345-3p in colorectal cancer (CRC) patients with liver metastasis. **A** Volcano plot of differentially expressed miRNAs in the GSE147603 dataset; (**B**) Relative expression of miR-345-3p in serum samples from CRC patients and healthy controls; (**C**) Receiver operating characteristic (ROC) curve for miR-345-3p in diagnosing CRC; (**D**) Comparisons of miR-345-3p expression between patients with and without liver metastasis; (**E**) ROC curve for miR-345-3p in diagnosing liver metastasis. *** *P* < 0.001 vs. healthy controls (**B**)/non-CRLM (**C**)

In the differential expression analysis of the GEO dataset GSE147603, miR-345-3p expression was significantly downregulated in liver metastatic tissues compared to CRC tissues without liver metastasis (|Log2FC| > 0, *P* < 0.05) (Fig. 1A). After rigorous hemolysis exclusion, a total of 200 qualified serum samples were included for subsequent analysis. qRT-PCR results from clinical serum samples further confirmed that miR-345-3p expression levels were significantly reduced in CRC patients compared to healthy individuals (*P* < 0.001, Fig. 1B). Additionally, serum miR-345-3p expression was further downregulated in the CRLM group, showing markedly lower levels than those in non-CRLM group (*P* < 0.001, Fig. 1C). Through ROC curve analysis, serum miR-345-3p demonstrated high diagnostic efficacy for distinguishing healthy individuals from CRC patients with an area under the ROC curve (AUC) of 0.898 (Fig. 1D) and exhibited good discriminatory ability for predicting liver metastasis in CRC with an AUC of 0.835(Fig. 1E).

### Associations of miR-345-3p expression with clinicopathological factors and prognosis of CRC patients

**Fig. 2:**
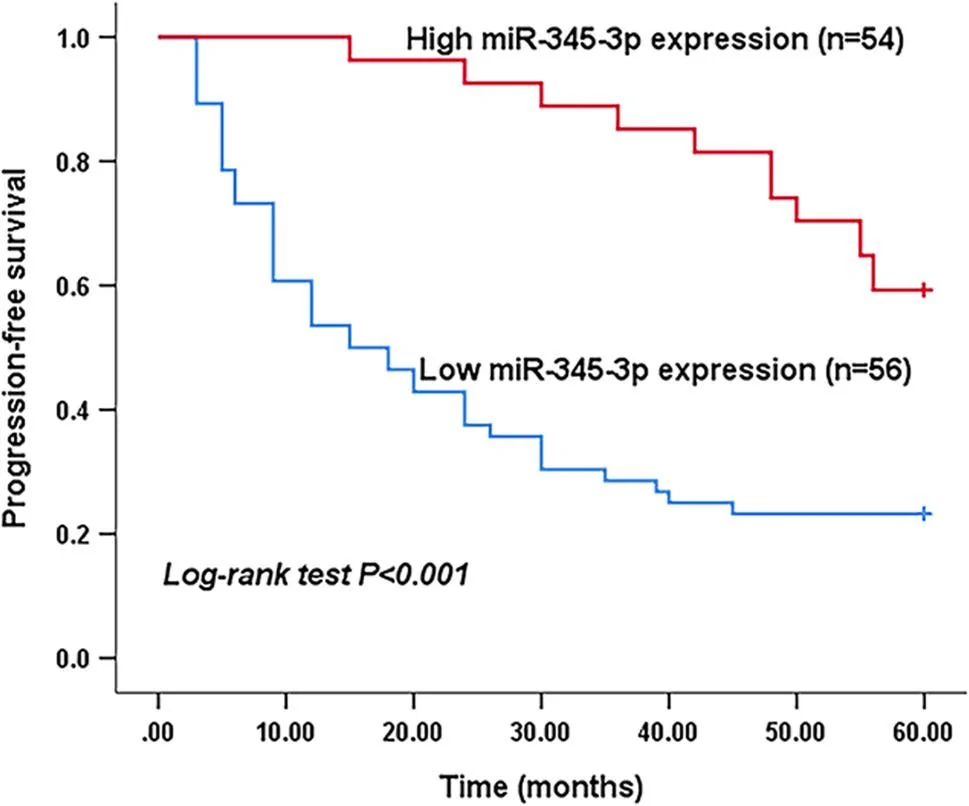
Survival analysis of colorectal cancer (CRC) patients based on miR-345-3p expression levels via Kaplan-Meier methods. Patients with low miR-345-3p expression (*n*=56) had significantly worse survival outcomes compared to those with high expression (*n*=54) (Log-rank test, *P* < 0.001)

Using the mean value of relative expression of miR-345-3p as the cutoff, the CRC cohort was stratified into two groups: low miR-345-3p (*n* = 56) and high miR-345-3p (*n* = 54). As indicated in Table 2, the Chi-square test revealed that abnormal miR-345-3p expression was closely associated with TNM stage (*P* = 0.002), depth of invasion (*P* = 0.032), and LNM (*P* = 0.032). Kaplan-Meier survival curves further demonstrated significantly inferior 5-year progression-free survival (PFS) in patients with low miR-345-3p expression compared to those with high miR-345-3p expression (log-rank test, *P* < 0.001, Fig. 2). Additionally, Cox multivariate analysis confirmed that low miR-345-3p expression was an independent predictor of unfavorable prognosis (Hazard ratio: HR = 0.306, 95% confidence interval: 95%CI = 0.178–0.525) (Table 3).

**Table 2:** Associations of miR-345-3p expression and clinicopathological features of CRC patients

Features	No. of cases (*n* = 110)	MiR-345-3p expression		*P* values
		Low (*n* = 56)	High (*n* = 54)	
Age (years)				0.424
< 60	63	30	33	
≥ 60	47	26	21	
Gender				0.686
Male	61	30	31	
Female	49	26	23	
Tumor locations				0.193
Colon	75	35	40	
Rectal	35	21	14	
Tumor size (cm)				0.073
< 5	66	29	37	
≥ 5	44	27	17	
Differentiation				0.098
Median-high	71	32	39	
Poor	39	24	15	
TNM stage				0.002
I-II	65	25	40	
III-IV	45	31	14	
Depth of invasion				0.025
T1-T2	70	30	40	
T3-T4	40	26	14	
LNM				0.032
Negative	84	38	46	
Positive	26	18	8	

*CRC* colorectal cancer, *TNM* Tumor Node Metastasis, *LNM* lymph node metastasis

*P* < 0.05 indicates a significant difference

**Table 3:** Cox regression analysis of miR-345-3p expression for overall survival in CRC patients

Characteristics	Univariate analysis		Multivariate analysis	
	HR (95%CI)	*P* value	HR (95%CI)	*P* value
Age (years)	1.343 (0.825–2.188)	0.236	/	/
Gender	1.046 (0.642–1.704)	0.856	/	/
Tumor locations	1.340 (0.806–2.230)	0.259	/	/
Tumor size (cm)	1.574 (0.966–2.567)	0.069	/	/
Differentiation	1.566 (0.955–2.568)	0.075	/	/
TNM stage	3.047 (1.853–5.011)	< 0.001	2.061 (1.183–3.591)	0.011
Depth of invasion	1.843 (1.125–3.019)	0.015	1.284 (0.760–2.171)	0.351
LNM	3.106 (1.857–5.195)	< 0.001	1.864 (1.026–3.388)	0.041
MiR-345-3p expression	0.264 (0.156–0.445)	< 0.001	0.306 (0.178–0.525)	< 0.001

*CRC* colorectal cancer, *TNM* Tumor Node Metastasis, *LNM* lymph node metastasis

*P* < 0.05 indicates a significant difference

### Logistic regression of risk factors for the occurrence of liver metastasis in CRC patients

To evaluate the risk factors for liver metastasis in CRC, logistic regression analysis was conducted. Univariate analysis revealed that TNM stage (Odds ratio: OR = 6.971, *P* = 0.001), LNM (OR = 5.427, *P* = 0.001), and low miR-345-3p expression (OR = 0.184, *P* = 0.004) were significantly associated with liver metastasis. Multivariate analysis further confirmed that low miR-345-3p expression (OR = 0.277, *P* = 0.043) is an independent risk factor for liver metastasis, along with TNM stage (OR = 3.946, *P* = 0.024) (Table 4).

**Table 4:** Logistic regression analysis of miR-345-3p for the risk for the occurrence of liver metastasis of CRC

Characteristics	Univariate analysis		Multivariate analysis	
	OR (95%CI)	*P* value	OR (95%CI)	*P* value
Age (years)	1.278 (0.492–3.319)	0.615	/	/
Gender	0.919 (0.352–2.399)	0.863	/	/
Tumor locations	1.089 (0.396–2.996)	0.868	/	/
Tumor size (cm)	2.375 (0.903–6.244)	0.079	/	/
Differentiation	2.396 (0.912–6.298)	0.076	/	/
TNM stage	6.971 (2.322–20.930)	0.001	3.946(1.202–12.950)	0.024
Depth of invasion	2.276 (0.868–5.969)	0.095	/	/
LNM	5.427 (1.956–15.056)	0.001	2.920(0.943–9.040)	0.063
MiR-345-3p expression	0.184 (0.057–0.590)	0.004	0.277(0.080–0.958)	0.043

*CRC* colorectal cancer, *TNM* Tumor Node Metastasis, *LNM* lymph node metastasis

*P* < 0.05 indicates a significant difference

### Impacts of miR-345-3p expression on cellular functions and EMT

**Fig. 3:**
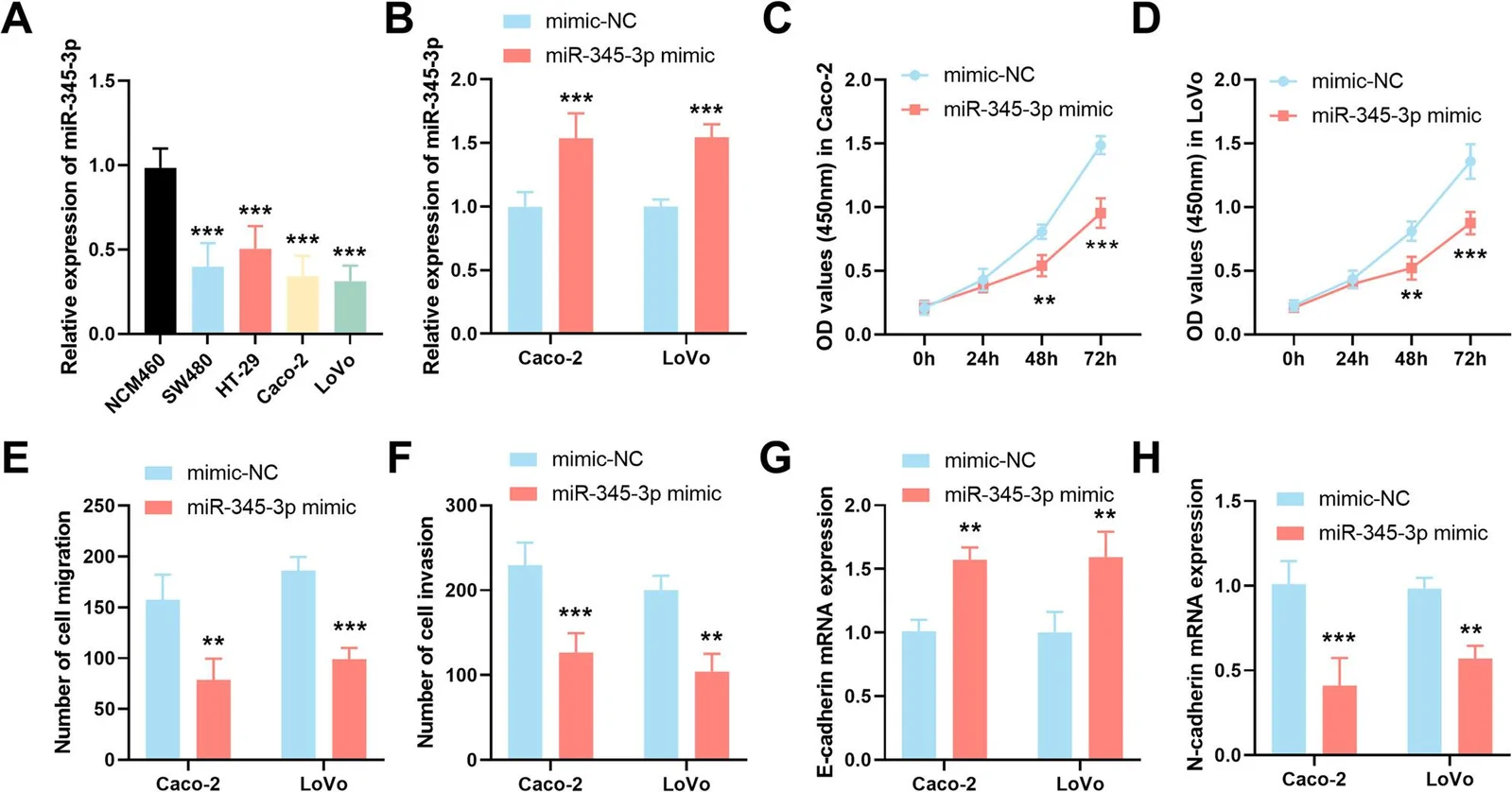
Effects of miR-345-3p on cellular functions and epithelial-mesenchymal transition (EMT) in colorectal cancer (CRC) cells. **A** Relative abundances of miR-345-3p in CRC cell lines (SW480, HT-29, Caco-2, LoVo) and normal colonic epithelial cell line NCM460. **B** Validation of transfection efficiency of miR-345-3p mimic in Caco-2 and LoVo cells. **C**-**D** Cell proliferation assessed in Caco-2 and LoVo cell after transfection. **E**-**F** Cell migration (**E**) and invasion (**F**) analyzed in CRC cells. **G**-**H** mRNA expression levels of E-cadherin and N-cadherin in CRC cells. Data are presented as mean±SD. ** *P* < 0.01 and *** *P* < 0.001 vs. mimic-NC

Compared to normal colonic epithelial cells (NCM460), all tested CRC cell lines (SW480, HT-29, Caco-2, and LoVo) exhibited significantly reduced expression of miR-345-3p (Fig. 3A). For further mechanistic studies, Caco-2 and LoVo models were employed. In vitro functional experiments demonstrated that transfecting Caco-2 and LoVo cells with miR-345-3p mimic significantly increased its endogenous expression (*P* < 0.001, Fig. 3B). Increased miR-345-3p expression notably suppressed the proliferation capacity of both cell lines (Fig. 3C-D). Moreover, cell migration (Fig. 3E) and invasion abilities were significantly attenuated in the miR-345-3p mimic group compared to the mimic-NC group (Fig. 3F). These in vitro data suggest that miR-345-3p suppresses the migratory and invasive potential of CRC cells, which are key steps in the metastatic cascade. Furthermore, miR-345-3p mimic treatment increased E-cadherin mRNA expression (Fig. 3G) while decreasing N-cadherin mRNA expression (Fig. 3H).

### Bioinformation analysis for miR-345-3p-related targets

**Fig. 4:**
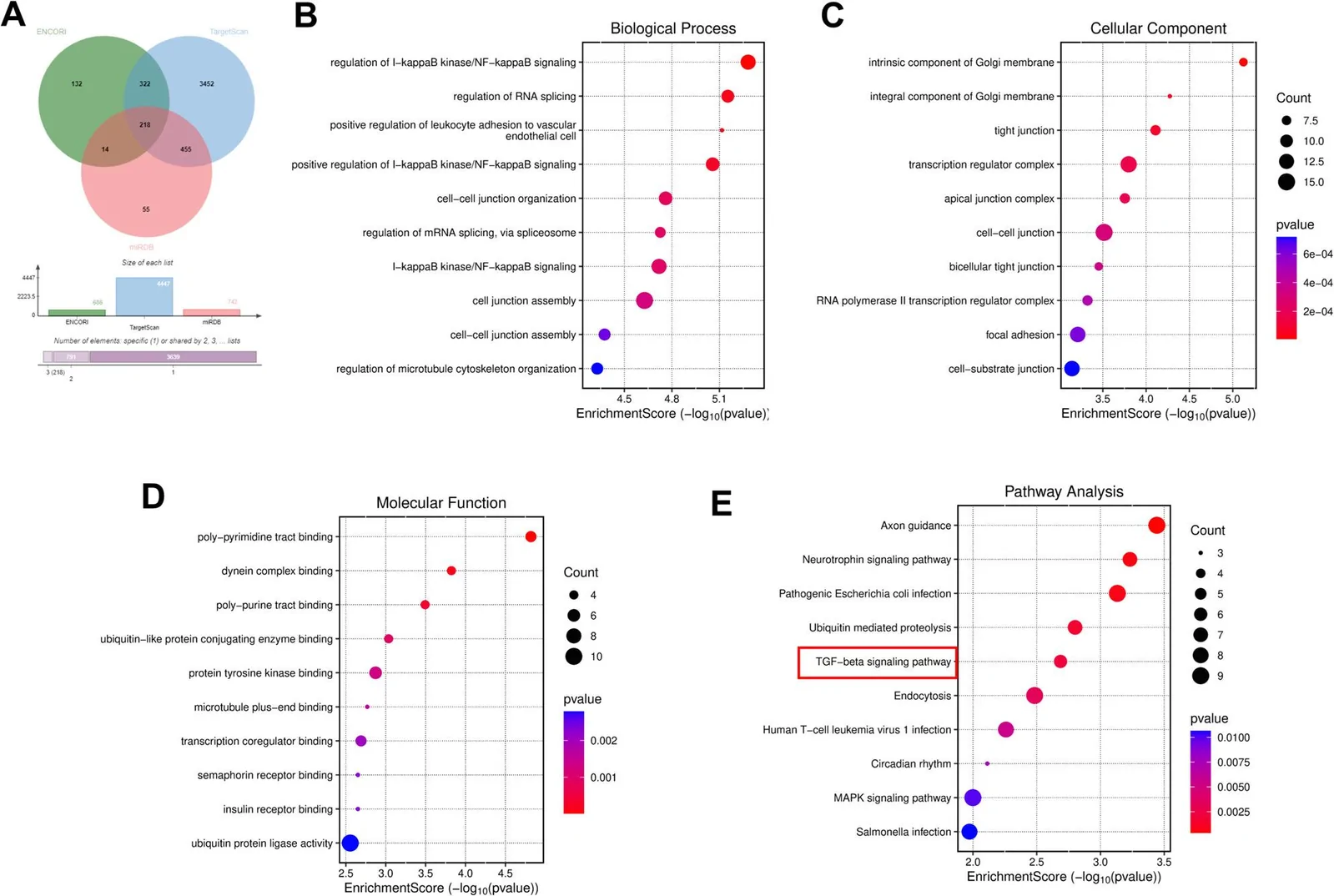
Bioinformatics analysis of potential target genes of miR-345-3p. **A** Venn diagram showing the overlapping target genes predicted by three databases: ENCORI, TargetScan, and miRDB. **B**-**D** Gene ontology (GO) enrichment analysis of the overlapping target genes in terms of biological process (**B**), cellular component (**C**), and molecular function (**D**). **E** Kyoto Encyclopedia of Genes and Genomes (KEGG) pathway enrichment of the overlapping target genes. The red box indicated to be selected for the subsequent experiments

To elucidate the underlying mechanism of miR-345-3p in CRC, its downstream target genes were predicted using bioinformatics methods. By integrating three public databases, namely ENCORI, TargetScan, and miRDB, we identified 218 overlapping target genes (Fig. 4A). GO functional enrichment analysis revealed that, within biological processes (BP), the target genes were significantly enriched in processes such as regulation of I-κB kinase/NF-κB signaling pathway and regulation of RNA splicing (Fig. 4B). At the cellular component (CC) level, they predominantly concentrated intrinsic component of Golgi membrane and tight junction (Fig. 4C). In the molecular function (MF) category, the primary functions included poly-pyrimidine tract binding and dynein complex binding (Fig. 4D). Furthermore, KEGG pathway enrichment analysis indicated significant enrichment of target genes in multiple pathways closely associated with tumor metastasis, including the TGF-β signaling pathway (Fig. 4E). Based on these findings, subsequent experiments in this study will focus on validating the role of TGF-β signaling pathway.

### Target relationship between miR-345-3p and KHDRBS1

**Fig. 5:**
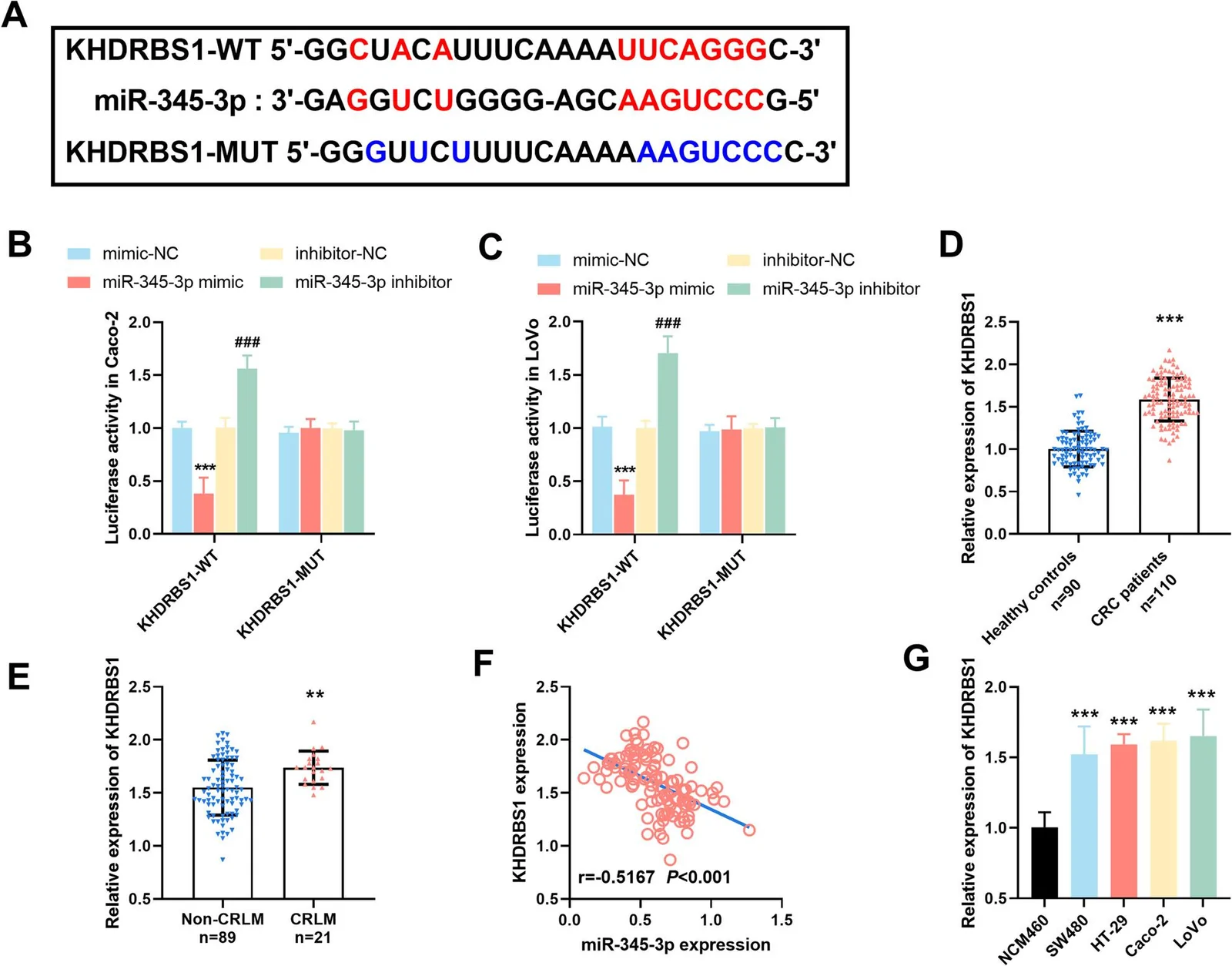
KH RNA binding domain containing, signal transduction associated 1 (KHDRBS1) is a direct target of miR-345-3p. **A** Schematic diagram of the predicted binding sites between miR-345-3p and KHDRBS1-WT or KHDRBS1-MUT 3’UTR of KHDRBS1. **B**-**C** Dual-luciferase reporter assay in colorectal cancer (CRC) cells co-transfected with the wide or mutant type and miR-345-3p mimic/inhibitor. **D** Relative miRNA expression levels of KHDRBS1 in serum samples from healthy controls and CRC patients. **E** A significant increase in serum KHDRBS1 levels in CRC patients with liver metastasis (CRLM) or without liver metastasis (non-CRLM). **F** Correlation analysis between the expression levels of miR-345-3p and KHDRBS1 in serum samples of CRC patients. **G** Relative abundances of KHDRBS1 mRNA in normal colon epithelial cells (NCM460) and CRC cell lines (SW480, HT-29, Caco-2, LoVo). Data were presented as mean±standard deviation (SD). ** *P* < 0.01 and *** *P* < 0.001 vs. mimic-NC (**B**), healthy controls (**D**), non-CRLM (**E**), and NCM460 (**G**); ### *P* < 0.001 vs. inhibitor-NC

To validate whether KHDRBS1 is directly targeted by miR-345-3p, a luciferase reporter assay was conducted. Figure 5A displayed the predicted binding sequences of miR-345-3p with the 3’ UTR region of KHDRBS1 mRNA, along with the designed mutation sites. In Caco-2 and LoVo cells, co-transfection with the miR-345-3p mimic significantly suppressed luciferase activity in the wild-type KHDRBS1 3’UTR reporter plasmid; conversely, transfection with the miR-345-3p inhibitor markedly enhanced luciferase activity (Figs. 5B-C). When the binding site in the KHDRBS1 3’UTR was mutated, the regulatory effects of miR-345-3p on reporter activity were abolished.

Clinical sample validation demonstrated a significant upregulation of KHDRBS1 mRNA expression in serum specimens from CRC patients compared to healthy individuals (*P* < 0.001, Fig. 5D), with even higher expression levels observed in patients with liver metastasis (Fig. 5E). Correlation analysis indicated a strong negative correlation between miR-345-3p and KHDRBS1 expression (*r* = -0.5167, *P* < 0.001, Fig. 5F). Additionally, KHDRBS1 expression was found to be elevated in CRC cell lines compared to NCM460 (Fig. 5G).

### Overexpression of KHDRBS1 reversed the tumor suppressive effect of miR-345-3p

**Fig. 6:**
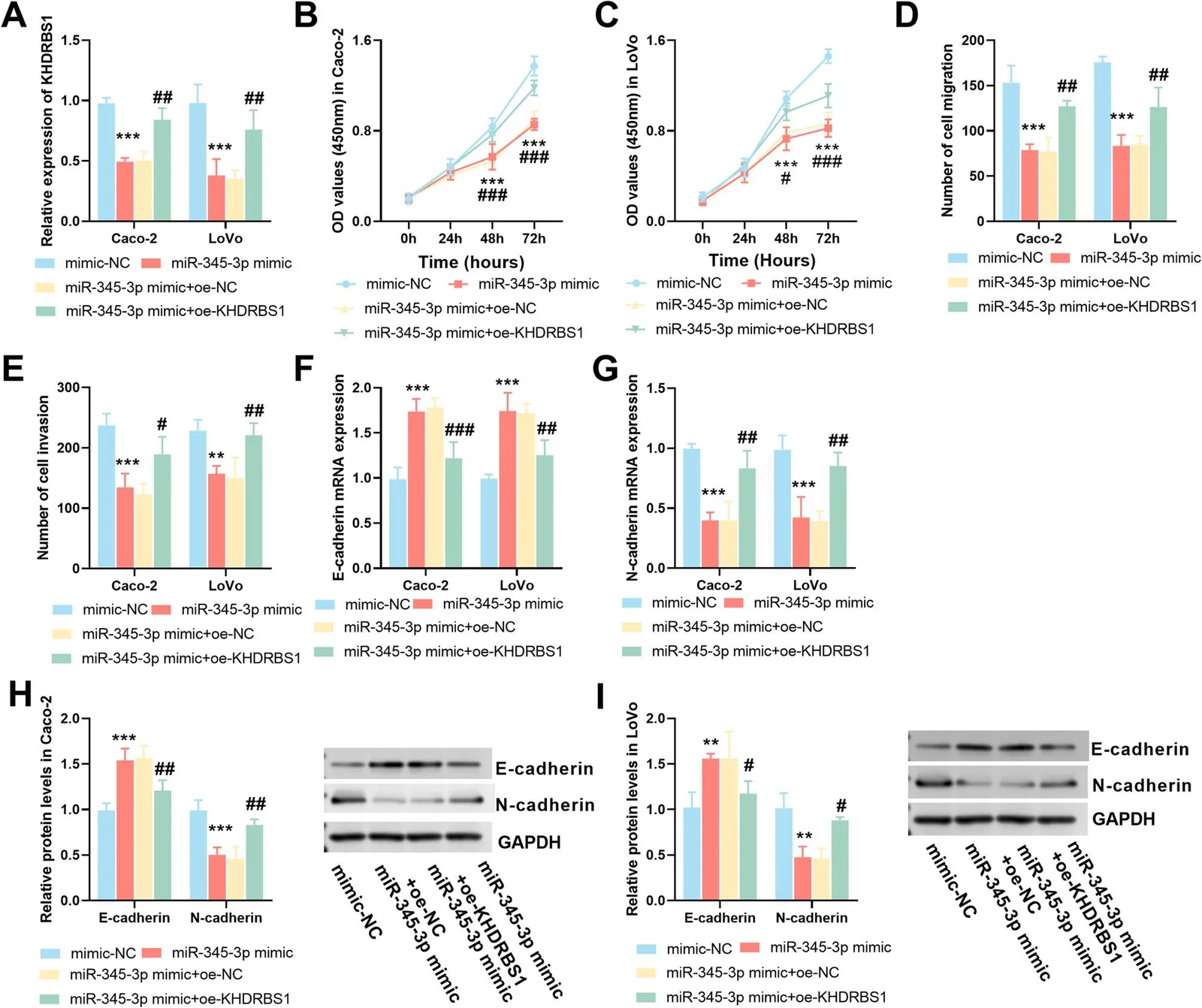
Overexpression of KH RNA binding domain containing, signal transduction associated 1 (KHDRBS1) rescues the tumor-suppressive effects of miR-345-3p in CRC cells. **A** Relative mRNA expression of KHDRBS1 in Caco-2 and LoVo cells after different transfections. **B**-**C** Cell proliferation was assessed in Caco-2 and LoVo cells after co-transfection of miR-345-3p and KHDRBS1. **D**-**E** Following co-transfection of miR-345-3p and KHDRBS1, cell migration (**D**) and invasion (**E**) were analyzed in CRC cells. **F**-**G** mRNA expression levels of E-cadherin and N-cadherin were determined in CRC cells. **H**-**I** Western blot analysis of E-cadherin and N-cadherin protein expression in Caco-2 and LoVo cells after co-transfection with miR-345-3p mimic and oe-KHDRBS1 or oe-NC. GAPDH served as a loading control. Data were expressed as mean±standard deviation (SD). ** *P* < 0.001 and *** *P* < 0.001 vs. mimic-NC, # *P* < 0.05, ## *P* < 0.01 and ### *P* < 0.001 vs. miR-345-mimic+oe-NC

To investigate whether miR-345-3p exerts its tumor-suppressive effects by targeting KHDRBS1, a rescue assay was conducted. As illustrated in Fig. 6A, co-transfection of a miR-345-3p mimic with a KHDRBS1 overexpression plasmid significantly restored KHDRBS1 expression levels in Caco-2 and LoVo cells compared to the miR-345-3p mimic group. Functional recovery experiments confirmed that KHDRBS1 overexpression effectively reversed the inhibitory effects on cell proliferation (Figs. 6B-C), migration (Fig. 6D), and invasion (Fig. 6E) induced by the overexpression of miR-345-3p. Furthermore, detection of EMT markers revealed that the restored KHDRBS1 expression partially counteracted the miR-345-3p-induced upregulation of E-cadherin and downregulation of N-cadherin (Fig. 6F-G). Consistent with the mRNA results, Western blot analysis confirmed that KHDRBS1 overexpression effectively reversed the miR-345-3p-induced upregulation of E-cadherin protein and downregulation of N-cadherin protein in both Caco-2 (Fig. 6H) and LoVo (Fig. 6I) cells.

### MiR-345-3p exerted its effects via modulating the TGF-β signaling pathway

**Fig. 7:**
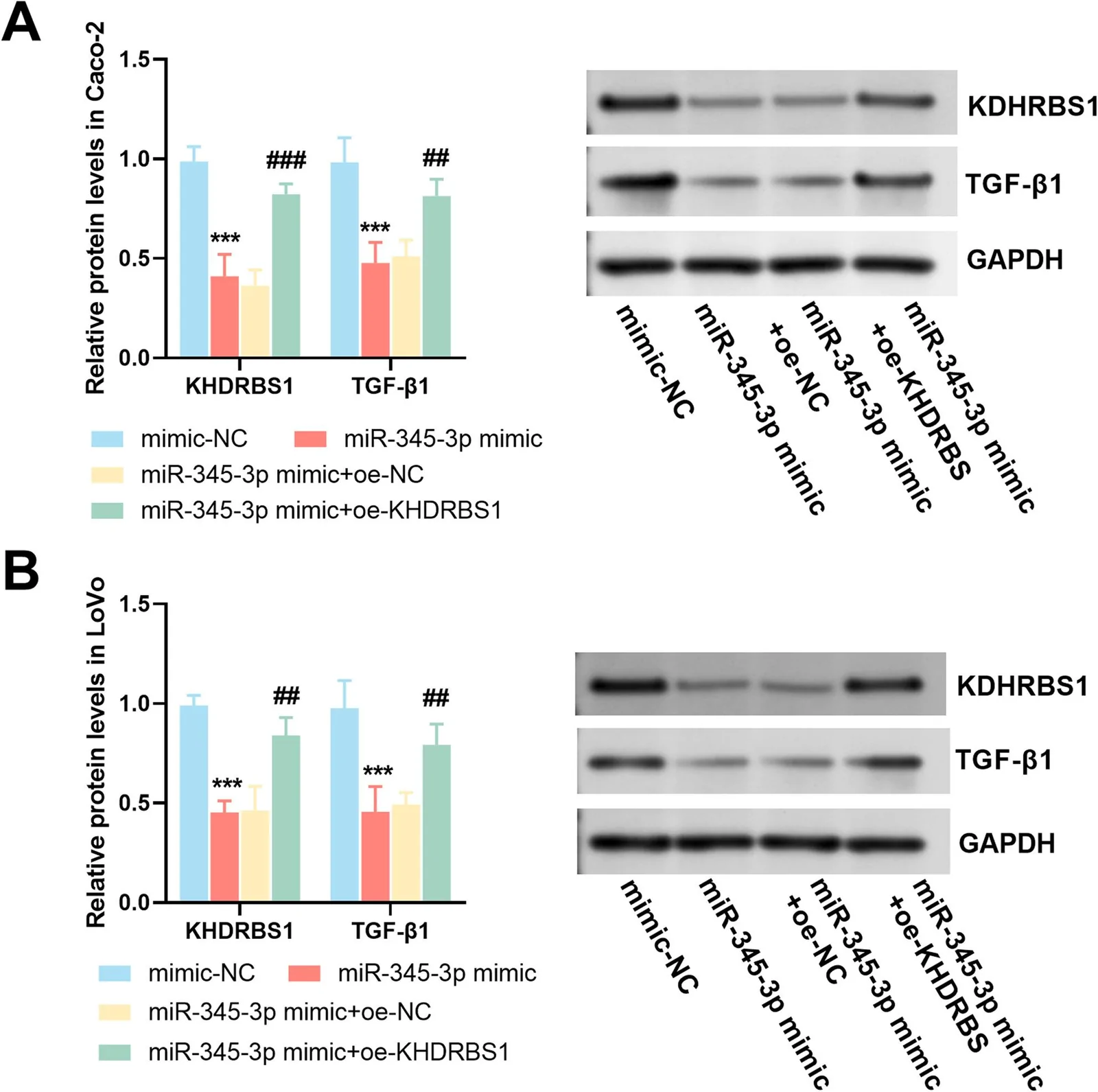
MiR-345-3p regulates the transforming growth factor-β (TGF-β) signaling pathway by targeting KH RNA binding domain containing, signal transduction associated 1 (KHDRBS1). **A** KHDBS1 and TGFβ1 protein expression levels in Caco-2 cells after different transfection conditions. Representative blots are presented with GAPDH serving as a loading control. **B** the protein levels of KHDBS1 and TGF-β1 in LoVo cells under different treatments. GAPDH served as a loading control and representative blots are shown. ** *P* < 0.01 and *** *P* < 0.001 vs. mimic-NC, # *P* < 0.05, ## *P* < 0.01 and ### *P* < 0.001 vs. miR-345-3p mimic+oe-NC

To further investigate the potential signaling pathways through which the miR-345-3p/KHDRBS1 axis influences CRC metastasis, Western blot analysis was performed. The overexpression of miR-345-3p in Caco-2 (Fig. 7A) and LoVo (Fig. 7B) cells significantly downregulated the protein expression of its target gene KHDRBS1, which was accompanied by a reduction in the expression levels of TGF-β1, a key factor in the TGF-β signaling pathway. Further rescue experiments demonstrated that co-transfection of the miR-345-3p mimic with KHDRBS1 overexpression plasmid restored KHDRBS1 protein expression and correspondingly increased TGF-β1 protein expression, partially reversing the inhibitory effect of miR-345-3p (Fig. 7).

## Discussion

This study systematically revealed the tumor-suppressive function of miR-345-3p in suppressing CRC cell migration and invasion in vitro, along with its molecular mechanisms through integrated bioinformatics analysis, validation of clinical samples, and in vitro functional experiments. These findings suggest a potential role for miR-345-3p in CRC liver metastasis, which warrants further in vivo validation. Moreover, serum levels of miR-345-3p were remarkably down-regulated in CRC patients with liver metastasis and were closely associated with adverse clinical and pathological features, shorter survival and a higher risk of liver metastasis. Functionally, miR-345-3p inhibited CRC cell growth and EMT progression. Mechanistic studies indicated that KHDRBS1 was a functional target of miR-345-3p and that the miR-345-3p/KHDRBS1 axis exerted its biological effects by regulating the TGF-β signaling pathway.

MiRNAs are pivotal modulator of gene expression in post-transcriptional activities. They play a dual role in tumorigenesis and progression, exhibiting highly tissue- and cancer-type-specific functions. This study reveals that miR-345-3p exerts tumor-suppressive effects in CRC, consistent with its reported roles in HCC [20], diffuse large B-cell lymphoma [25], and glioblastoma [26]. However, other studies indicate that miR-345-3p is widely detected in breast cancer [21] and oral squamous cell carcinoma [27], where it promotes tumor progression. This apparent functional discrepancy suggests that miRNA activity is highly dependent on its cellular environment and the specific target gene networks it regulates. Our study provides new evidence for the tumor-suppressing function of miR-345-3p in gastrointestinal tumors, particularly CRC.

Utilizing multi-database cross-prediction and dual luciferase reporter assays, we demonstrated for the first time that KHDRBS1 (Sam68) is a key target gene of miR-345-3p in CRC. KHDRBS1, an RNA-binding protein involved in mRNA alternative splicing, stability, and translational regulation, has been shown to promote tumorigenesis in various human tumors, including CRC [22, 28, 29]. The present study corroborates the hypothesis that KHDRBS1 expression is elevated in the serum of CRC patients, demonstrating a significant inverse correlation between miR-345-3p levels and the occurrence of liver metastasis. Further experiments in rescue research indicated that KHDRBS1 overexpression effectively reversed the tumor-suppressive phenotype of miR-345-3p, confirming that miR-345-3p primarily functions by targeting and inhibiting KHDRBS1.

At the mechanistic level, KEGG enrichment analysis indicated that the TGF-β pathway is a key pathway potentially regulated by miR-345-3p. This pathway has been observed to exhibit dual roles in tumor progression [30, 31]. In the early stages of tumorigenesis, it serves a tumor-suppressive function; however, during later stages, it promotes metastatic progression through mechanisms such as EMT induction and immune escape [32]. Experimental results confirmed that miR-345-3p could downregulate KHDRBS1 expression and the protein levels of TGF-β1, a pivotal molecule in the TGF-β pathway. Furthermore, the restoration of KHDRBS1 expression led to a partial reversal of this inhibitory effect. These findings suggested that miR-345-3p might exert an inverse regulatory impact on the TGF-β pathway by targeting KHDRBS1, thereby suppressing the invasive and metastatic capacity of CRC. This study established a connection between the miR-345-3p/KHDRBS1 axis and a well-established pro-metastatic pathway offering a novel perspective on understanding the molecular mechanisms underlying liver metastasis in CRC. It is important to be note that the mechanistic link between KHDRBS1 and TGF-β1 remains indirect. As an RNA-binding protein, KHDRBS1 likely modulates TGF-β1 expression through intermediate factors rather than via direct promoter binding. Future mechanistic studies are necessary to clarify this relationship.

The clinical significance of this study lies in its proposal that circulating miR-345-3p may serve as a potential non-invasive biomarker for predicting liver metastasis in CRC and assessing prognosis. ROC curve analysis demonstrated its strong diagnostic value. Combined with multifactorial regression analysis results, decreased miR-345-3p expression was confirmed as an independent risk factor influencing liver metastasis and poor clinical outcome in CRC patients. This provides a novel reference indicator for the clinical identification of high-risk metastatic patients and enables early intervention.

The present study acknowledges several limitations. Firstly, the primary limitation is the absence of tumor tissue samples. All clinically relevant analyses were conducted using serum samples. While serum miRNAs serve as valuable liquid biopsy biomarkers, they do not directly reflect the molecular events occurring the tumor microenvironment. Consequently, the expression levels of miR-345-3p in CRC tumor tissues and its negative correlation with KHDRBS1 require confirmation in future tissue cohort studies. Secondly, the lack of in vivo animal model experiments means that the conclusion regarding the potential role of miR-345-3p in liver metastasis is primarily derived from in vitro cellular functional assays assessing migration and invasion. Tumor metastasis is a complex process involving multiple steps and interactions among various cell types, and in vitro experiments cannot fully replicate the in vivo microenvironment. Therefore, the potential inhibitory effect of miR-345-3p on liver metastasis in vivo necessitates further validation through animal model studies. Thirdly, the sample size for the liver metastasis subgroup is relatively small, which may introduce selection bias. Thus, the conclusions of this study require further validation in larger, multicenter prospective cohorts. Finally, additional investigation is needed to elucidate the specific molecular mechanisms by which miR-345-3p modulates the TGF-β signaling pathway. This includes determining whether KHDRBS1 directly influences TGF-β1 transcription and translation or acts indirectly by modulating its upstream or downstream molecules. It is also important to note that the cutoff for stratifying patients into high and low miR-345-3p expression groups was based on the cohort mean, representing an exploratory rather than a clinically validated threshold. While this approach is commonly employed in discovery-phase studies, future large-scale, multicenter prospective cohorts will be essential to establish an optimized and externally validated cutoff for clinical application.

In summary, our study demonstrates that decreased levels of circulating miR-345-3p are associated with liver metastasis in CRC. In vitro experiments indicate that miR-345-3p may regulate the TGF-β signaling pathway by targeting KHDRBS1, thereby suppressing the EMT-related protein and mRNA expression changes and the migratory and invasive capabilities of CRC cells. These findings suggest a potential role for miR-345-3p in CRC liver metastasis, but in vivo studies are needed to confirm this effect.

## Data Availability

The datasets used and/or analysed during the current study are available from the corresponding author on reasonable request.

## References

[1] Baidoun F, Elshiwy K, Elkeraie Y, Merjaneh Z, Khoudari G, Sarmini MT, et al. Colorectal Cancer Epidemiology: Recent Trends and Impact on Outcomes. Curr Drug Targets. 2021;22(9):998–1009. 33208072 10.2174/1389450121999201117115717

[2] Liu SC, Zhang H. Early diagnostic strategies for colorectal cancer. World J Gastroenterol. 2024;30(33):3818–22. 39351429 10.3748/wjg.v30.i33.3818 PMC11438623

[3] Li Q, Geng S, Luo H, Wang W, Mo YQ, Luo Q, et al. Signaling pathways involved in colorectal cancer: pathogenesis and targeted therapy. Signal Transduct Target Ther. 2024;9(1):266. 39370455 10.1038/s41392-024-01953-7 PMC11456611

[4] Peng G, Zhong L, Luo L, Ju Y, Lu Y, Ng L, et al. Cancer cell SPOCD1 promotes colorectal cancer liver metastasis by activating the CXCL12/CXCR4 signaling pathway in cancer-associated fibroblasts. J Transl Med. 2025;23(1):902. 40804742 10.1186/s12967-025-06863-y PMC12351940

[5] Song IH, Lee SB, Jeong BK, Park J, Kim H, Lee G, et al. T cell receptor clonotype in tumor microenvironment contributes to intratumoral signaling network in patients with colorectal cancer. Immunol Res. 2024;72(5):921–37. 39112913 10.1007/s12026-024-09478-5

[6] Ionescu VA, Gheorghe G, Bacalbasa N, Chiotoroiu AL, Diaconu C. Colorectal Cancer: From Risk Factors to Oncogenesis. Med (Kaunas). 2023;59(9):1646. 10.3390/medicina59091646 PMC10537191 37763765

[7] Patel SG, Karlitz JJ, Yen T, Lieu CH, Boland CR. The rising tide of early-onset colorectal cancer: a comprehensive review of epidemiology, clinical features, biology, risk factors, prevention, and early detection. Lancet Gastroenterol Hepatol. 2022;7(3):262–74. 35090605 10.1016/S2468-1253(21)00426-X

[8] Zhang N, Ng AS, Cai S, Li Q, Yang L, Kerr D. Novel therapeutic strategies: targeting epithelial-mesenchymal transition in colorectal cancer. Lancet Oncol. 2021;22(8):e358–68. 34339656 10.1016/S1470-2045(21)00343-0

[9] Han L, Wang S, Wei C, Fang Y, Huang S, Yin T, et al. Tumour microenvironment: a non-negligible driver for epithelial-mesenchymal transition in colorectal cancer. Expert Rev Mol Med. 2021;23:e16. 34758892 10.1017/erm.2021.13

[10] Ni Q, Li M, Yu S. Research Progress of Epithelial-mesenchymal Transition Treatment and Drug Resistance in Colorectal Cancer. Technol Cancer Res Treat. 2022;21:15330338221081219. 35435774 10.1177/15330338221081219 PMC9019367

[11] Shin AE, Giancotti FG, Rustgi AK. Metastatic colorectal cancer: mechanisms and emerging therapeutics. Trends Pharmacol Sci. 2023;44(4):222–36. 36828759 10.1016/j.tips.2023.01.003 PMC10365888

[12] Liu X, Wang X, Yang Q, Luo L, Liu Z, Ren X, et al. Th17 Cells Secrete TWEAK to Trigger Epithelial-Mesenchymal Transition and Promote Colorectal Cancer Liver Metastasis. Cancer Res. 2024;84(8):1352–71. 38335276 10.1158/0008-5472.CAN-23-2123

[13] Ruan Y, Lu G, Yu Y, Luo Y, Wu H, Shen Y, et al. PF-04449913 Inhibits Proliferation and Metastasis of Colorectal Cancer Cells by Down-regulating MMP9 Expression through the ERK/p65 Pathway. Curr Mol Pharmacol. 2024;17:e150923221164. 37724680 10.2174/1874467217666230915125622

[14] Ge H, Yan Y, Wang H, Bian J, Deng Z, Su X, et al. Circular RNA hsa_circ_0005939 Regulates UHRF1BP1L Expression by Targeting miR-4693-3p to Promote Colorectal Cancer Progression. Protein Pept Lett. 2024;31(6):437–46. 38918974 10.2174/0109298665297110240611115010

[15] Wu M, Wu X, Han J. KIF20A Promotes CRC Progression and the Warburg Effect through the C-Myc/HIF-1alpha Axis. Protein Pept Lett. 2024;31(2):107–15. 38037834 10.2174/0109298665256238231120093150

[16] Tian S, Chen M, Jing W, Meng Q, Wu J. miR-1204 Positioning in 8q24.21 Involved in the Tumorigenesis of Colorectal Cancer by Targeting MASPIN. Protein Pept Lett. 2024;31(7):544–58. 39082173 10.2174/0109298665305114240718072029

[17] Shah H, Khan NA, Ullah MI, Khan UZ, Irfan U, Ahmad I. Comparative Efficacy of Thermal Ablation and Surgical Resection in Patients with Colorectal Cancer Liver Metastasis: A Systematic Review and Meta-analysis. J Gastrointest Cancer. 2025;56(1):188. 40946121 10.1007/s12029-025-01314-9

[18] Hussen BM, Hidayat HJ, Salihi A, Sabir DK, Taheri M, Ghafouri-Fard S. MicroRNA: A signature for cancer progression. Biomed Pharmacother. 2021;138:111528. 33770669 10.1016/j.biopha.2021.111528

[19] Huang X, Zhu X, Yu Y, Zhu W, Jin L, Zhang X, et al. Dissecting miRNA signature in colorectal cancer progression and metastasis. Cancer Lett. 2021;501:66–82. 33385486 10.1016/j.canlet.2020.12.025

[20] Xu K, Xia P, Gongye X, Zhang X, Ma S, Chen Z, et al. A novel lncRNA RP11-386G11.10 reprograms lipid metabolism to promote hepatocellular carcinoma progression. Mol Metab. 2022;63:101540. 35798238 10.1016/j.molmet.2022.101540 PMC9287641

[21] Zeng Q, Jin F, Qian H, Chen H, Wang Y, Zhang D, et al. The miR-345-3p/PPP2CA signaling axis promotes proliferation and invasion of breast cancer cells. Carcinogenesis. 2022;43(2):150–9. 34922339 10.1093/carcin/bgab124 PMC9272193

[22] Wang X, Yu H, Sun W, Kong J, Zhang L, Tang J, et al. The long non-coding RNA CYTOR drives colorectal cancer progression by interacting with NCL and Sam68. Mol Cancer. 2018;17(1):110. 30064438 10.1186/s12943-018-0860-7 PMC6069835

[23] Kirschner MB, Kao SC, Edelman JJ, Armstrong NJ, Vallely MP, van Zandwijk N, et al. Haemolysis during sample preparation alters microRNA content of plasma. PLoS ONE. 2011;6(9):e24145. 21909417 10.1371/journal.pone.0024145 PMC3164711

[24] Blondal T, Jensby Nielsen S, Baker A, Andreasen D, Mouritzen P, Wrang Teilum M, et al. Assessing sample and miRNA profile quality in serum and plasma or other biofluids. Methods. 2013;59(1):S1–6. 23036329 10.1016/j.ymeth.2012.09.015

[25] Li Y, Lv Y, Wang J, Zhu X, Chen J, Zhang W, et al. LncRNA NORAD Mediates the Proliferation and Apoptosis of Diffuse Large-B-Cell Lymphoma via Regulation of miR-345-3p/TRAF6 Axis. Arch Med Res. 2022;53(3):271–9. 35164979 10.1016/j.arcmed.2022.01.004

[26] Cao J, Tang Z, Su Z. Long non-coding RNA LINC01426 facilitates glioblastoma progression via sponging miR-345-3p and upregulation of VAMP8. Cancer Cell Int. 2020;20:327. 32699526 10.1186/s12935-020-01416-3 PMC7372762

[27] Scholtz B, Horvath J, Tar I, Kiss C, Marton IJ. Salivary miR-31-5p, miR-345-3p, and miR-424-3p Are Reliable Biomarkers in Patients with Oral Squamous Cell Carcinoma. Pathogens. 2022;11(2):229. 10.3390/pathogens11020229 PMC8876825 35215172

[28] Wang L, Cui Y, Liao W, Liu S. [Role of Sam68 in proliferation, invasion and migration of colorectal cancer cells in vitro]. Nan Fang Yi Ke Da Xue Xue Bao. 2014;34(4):546–51. 24752106

[29] Fan R, Liu F, Gong Q, Liu D, Tang S, Shen D. KHDRBS1 as a novel prognostic signaling biomarker influencing hepatocellular carcinoma cell proliferation, migration, immune microenvironment, and drug sensitivity. Front Immunol. 2024;15:1393801. 38660302 10.3389/fimmu.2024.1393801 PMC11041018

[30] Lai XN, Li J, Tang LB, Chen WT, Zhang L, Xiong LX. MiRNAs and LncRNAs: Dual Roles in TGF-beta Signaling-Regulated Metastasis in Lung Cancer. Int J Mol Sci. 2020;21(4):1193. 10.3390/ijms21041193 PMC7072809 32054031

[31] Henriques A, Salvany-Celades M, Nieto P, Palomo-Ponce S, Sevillano M, Hernando-Momblona X, et al. TGF-beta builds a dual immune barrier in colorectal cancer by impairing T cell recruitment and instructing immunosuppressive SPP1(+) macrophages. Nat Genet. 2025;57(12):3050–65. 41203813 10.1038/s41588-025-02380-2

[32] Wang J, Xu Z, Wang Z, Du G, Lun L. TGF-beta signaling in cancer radiotherapy. Cytokine. 2021;148:155709. 34597918 10.1016/j.cyto.2021.155709

